# Tentative Approaches for Extraction of Lanthanides from Wastewater with Low Metal Concentration

**DOI:** 10.3390/membranes13050467

**Published:** 2023-04-27

**Authors:** José M. Carretas, Luís M. Ferreira, Pedro M. P. Santos, Susana S. Gomes, Maria Fátima Araújo, Leonor Maria, João Paulo Leal

**Affiliations:** 1 Centro de Química Estrutural (CQE), Instituto Superior Técnico (IST), Universidade de Lisboa, Campus Tecnológico e Nuclear, EN 10, 2695-066 Bobadela, Portugal; carretas@ctn.tecnico.ulisboa.pt (J.M.C.);; 2 Departamento de Engenharia e Ciências Nucleares (DECN), Instituto Superior Técnico (IST), Universidade de Lisboa, Campus Tecnológico e Nuclear, EN 10, 2695-066 Bobadela, Portugal; ferreira@ctn.tecnico.ulisboa.pt (L.M.F.);; 3 Centro de Ciências e Tecnologias Nucleares (C2TN), Instituto Superior Técnico (IST), Universidade de Lisboa, Campus Tecnológico e Nuclear, EN 10, 2695-066 Bobadela, Portugal

**Keywords:** circular economy, metal removal, wastewater, lanthanides

## Abstract

Lanthanides are critical elements, and their recovery from wastewater increases the availability of these elements and reduces their impacts on the environment. In this study, tentative approaches to extract lanthanides from low-concentration aqueous solutions were investigated. PVDF membranes soaked with different active compounds or synthesized chitosan-based membranes containing these active compounds were used. The membranes were immersed in 10^−4^ M of aqueous solutions of selected lanthanides, and their extraction efficiency was assessed using ICP-MS. The PVDF membranes showed quite poor results, with only the membrane with oxamate ionic liquid giving some positive results (0.75 mg of Yb, 3 mg of lanthanides per gram of membrane). However, the chitosan-based membranes led to very interesting results, with the maximum concentration factor for the final solution relative to the initial solution being 13 times higher for Yb, which was obtained with the chitosan–sucrose–citric acid membrane. Several of the chitosan membranes, namely the one with 1-Butyl-3-methylimidazolium-di-(2-ethylhexyl)-oxamate, could extract around 10 mg of lanthanides per gram of membrane, with the better one being the membrane with sucrose/citric acid that achieved more than 18 mg/g of membrane. The use of chitosan for this purpose is a novelty. Since these membranes are easily prepared and have a very low cost, practical applications can be envisaged after further studies to better understand the underlying mechanism.

## 1. Introduction

Rare-earth elements are a group of 17 specialty metals including Sc, Y, and 15 lanthanides. While rare-earth elements (and lanthanides) are common in the Earth’s crust, they tend to be very difficult to extract in usable quantities. Thus, they are not considered rare because they are hard to find but because it is difficult to extract enough of the pure form of each element to meet industrial needs. Lanthanides have been widely used in a variety of fields, and their use is expected to increase significantly in near future [1]. With a large part of the world’s reserves and an overwhelming fraction of their production located in China, the European Union has defined lanthanides as Critical Raw Materials (CRM) in all four CRM lists produced (2011, 2014, 2017 and 2020) [2]. In 2020, the European Commission launched the European Raw Materials Alliance (ERMA), which first aim is to build resilience and strategic autonomy for Europe’s rare-earth metal and magnet value chains [3]. Recently, rare-earth elements (REEs) are discovered to be biologically significant, with the finding of proteins being specifically induced by the presence of lanthanides [4].

In conventional economy, materials are taken from the Earth’s crust, and the products manufactured from them are used for a certain amount of time and eventually thrown away as waste. The process is linear. In contrast, in a circular economy, the approach is different and is based on three principles: eliminate waste and pollution, circulate products and materials, and regenerate nature. Therefore, the economic activities of other values and challenges, such as climate change, biodiversity loss, waste, and pollution, are also considered. This concept has been around for at least a few decades, but only in recent years that it has gained global interest and momentum [5].

Innovative processes for treating industrial wastewater containing metals had been under study in recent years [6]. The focus of these studies is the removal of metals from industrial wastewater as a pure environmental protection measure. These approaches are very relevant, but the recent concept of “harvesting metals from wastewater” is a step further that has recently gained attention as part of a circular economic strategy in the water and wastewater sector model [7], and lanthanide economy is not an exception [8].

Ionic liquids (ILs) are liquid organic salts with some very interesting properties that allow their use in a variety of applications, which has prompted various researchers to investigate their utilization in various fields [9,10]. Usually, they are made of a voluminous organic cation and an organic or inorganic anion. The unsymmetrical and bulky structure of constituent ions contributes to making them different from conventional salts. They possess several peculiar properties, such as negligible vapor pressure, non-flammability, large range of viscosities and densities, high thermal stability, and wide electrochemical window, and they can be tuned for a precise purpose using an adequate choice of cation and anion. Ionic liquids have been used for some years as extractants of metals, namely metals with a high molecular weight, either by themselves or incorporated in membranes [11,12]. Examples of recently developed approaches for REE extraction and separation can be found in the literature, including chemical precipitation, ion exchange, solvent extraction, membrane separation, and adsorption [13,14,15,16], with the hydrometallurgical and pyrometallurgical methods being the main recovery routes [1]. Additionally, separation of REE by using ionic liquids is found in the literature [17,18]. Nevertheless, membranes are the best choice for very dilute solutions, with membranes that could concentrate metal being the choice to search for. Anyhow, due to the complicated equipment of the liquid membrane separation method and the low stability of membranes, it is difficult to use this method in industry [19]. Furthermore, the price of some ionic liquids may have prevented its more widespread use. These are likely the reasons why the results in the literature considering lanthanides and membranes are not abundant and studies conducted with membranes have mainly focused on the extraction of one single metal or the separation of two metals.

In 2010, Trtic-Petrovic et al. studied the extraction of lutetium using a single fiber-supported liquid membrane containing di-(2-ethylhexyl) phosphoric acid (DEHPA) [20]. The authors used a concentration of 1.1 × 10^−3^ M of lutetium and could achieve an efficiency removal of 99% at the best conditions.

Additionally, the separation selectivity of a mixture of yttrium, neodymium, and dysprosium was studied using simulation only, with bis-(2-ethylhexyl) hydrogen phosphate (D_2_EHPA) as an extractant in a supported liquid membrane [21].

In 2015, two supported liquid membrane systems with membranes impregnated with either di-(2-ethylhexyl) phosphoric acid (HDEHP) or tributyl phosphate (TBP) [22] were investigated. The HDEHP-impregnated membrane was used to extract neodymium (III), representing a typical trivalent lanthanide and cerium, which was oxidized by sodium bismuthate from a trivalent to a tetravalent state and then extracted by TBP. These two metals were used as surrogates of americium and curium (with the aim that the same systems could be used for their separation).

Finally, Asadollahzadeh et al. studied the extraction of cerium ions from an aqueous medium with the membrane extraction processes [23]. The studied PTFE membrane was loaded with imidazolium ionic liquid and organophosphorus extractants. The results show that [C_6_mim][NTf_2_] ionic liquid improves the extraction procedure.

An exception is probably the study by Dolezal et al., which covered several lanthanides along the entire series and used a supported kerosene membrane containing di-(2-ethylhexyl) phosphoric acid (DEHPA) as a carrier [24]. However, even in this case, each lanthanide was studied one at a time. In this work, two types of membranes were used: (1) PVDF membranes were soaked with ionic liquids or other active compounds, and (2) chitosan-based membranes were prepared with ionic liquids or other active compounds in their composition. In both cases, the goal was the same: to remove as much lanthanides as possible from low-concentration solutions and, ideally, to concentrate the final solution relative to the initial one, while performing this with all the studied metals present at the same time. In addition, to our best knowledge, this is the first time that chitosan-based membranes are used to extract lanthanides.

## 2. Materials and Methods

### 2.1. Materials/Reagents

All the chemicals and reagents used in this study were of analytical grade. Nitric acid (65%, Panreac, Barcelona, Spain), citric acid (>99.5%, Sigma Aldrich, Saint Louis, MO, USA), myristic acid (98%, Alfa Aesar, Haverhill, MA, USA), succinic acid (>99.5%, Sigma Aldrich, Saint Louis, MO, USA), and sucrose (Sidul, Santa Iria de Azoia, Portugal, >99.5%) were used as received. Lanthanide nitrates (Ce, Nd, Sm, Gd, Dy, Er, and Yb; 99.9%) were purchased from Sigma AldrichMO, USA. PVDF membranes from Immobilon (Ref. ISEQ07850, Merck, Germany) with a 0.2 µm pore size were used. Chitosan medium with a molecular weight of (1.9 × 10^5^ to 3.1 × 10^5^ Da) and 75–85% deacetylated was obtained from the Aldrich Chemical Company, Inc., Milwaukee, WI, USA.

To load or to synthesize the membranes, several ionic liquids and other compounds or mixtures were used. To keep it more simple and concise, especially in figures, a letter was assigned to each one of them: A—1-n-Butyl-3-methylimidazolium hexafluorophosphate ([Bmim][PF_6_]); B—trihexyltetradecylphosphonium bis(trifluoromethylsulfonyl)amide ([P_66614_][NTf_2_]); C—1-Butyl-3-methylimidazolium bis(trifluromethyl sulfonyl)imide ([C_4_mim][NTf_2_]); D—1-Butyl-3-methylimidazolium-di(2-ethylhexyl)-oxamate ([C_4_mim][DEHOX]); E—tributyl phosphate; F—sucrose/citric acid; G—sucrose/myristic acid; and H—sucrose/succinic acid.

Compounds A to C and E were purchased from Alfa Aesar (98%), Aldrich (95%), Aldrich (98%), and Sigma-Aldrich (99%), respectively, and used as supplied. Compound D was synthesized as described in reference [25]. Compounds F to H were synthesized as described below. Compounds F to H were mixtures of sucrose with carboxylic acid. The idea was to obtain Lewis adducts that could coordinate to the metals and still be fixed at the membranes.

### 2.2. Synthesis of Compound F

This compound was synthesized by adding 10.0005 g (0.05205 mol) of citric acid and 4.9974 g (0.01460 mol) of sucrose to 50 mL of distilled water. The mixture was placed in an oven at 50 °C for 6 days. The mixture always looked homogeneous, so homogenization was never needed. At the end, a dark brown viscous liquid with 4% (*w*/*w*) of water in its composition was obtained. Compound F was very stable: no change in appearance was observed after weeks of being kept in a closed flask.

### 2.3. Synthesis of Compound G

This compound was synthesized by adding 3.0066 g (0.01316 mol) of myristic acid and 7.6787 g (0.02243 mol) of sucrose to 50 mL of distilled water. The mixture was placed in an oven at 60 °C for 7 days. During this period, the mixture was homogenized every 24 h. At the end, a white viscous liquid that still possessed 6% (*w*/*w*) of water was obtained. Attempts to further dry the compound led to a white deposit in the liquid. After some weeks resting in a closed flask, some white deposit was detected, which disappeared with shaking or after slightly heating the flask.

### 2.4. Synthesis of Compound H

This compound was synthesized by adding 3.5038 g (0.029670 mol) of succinic acid and 12.0426 g (0.035181 mol) of sucrose to 50 mL of distilled water. The mixture was placed in an oven at 50 °C for 3 days. During this period, the mixture was homogenized every 24 h. At the end, a yellowish viscous liquid that still possessed 10% (*w*/*w*) of water was obtained. Attempts to further dry the compound led to a white deposit in the liquid. After some weeks resting in a closed flask, some white deposit was detected, which disappeared with shaking or after slightly heating the flask.

### 2.5. Preparation of Membranes of PVDF with Supported Active Compound

PVDF membranes from Immobilon (see above) with a 0.2 µm pore size were used. These membranes were chosen because they are hydrophobic and present a high resistance to solvents, namely water. Supported liquid membranes were prepared under vacuum via direct immersion of the membranes into 2 mL of the active compounds (or mixtures) for 1 h. Excess solution was removed with absorbent paper, and the impregnated membranes were stored overnight in an air desiccator with silica gel. To determine the amount of the loaded active compounds (or mixtures), the membranes were weighed before and after the impregnation procedure. The membranes used for impregnation were around 2 cm^2^.

### 2.6. Preparation of Chitosan Membranes

The preparation of the chitosan membranes was inspired by the procedures described in the literature [26,27]. In brief, 200 mg of chitosan in powder was placed in a glass. Then, a variable amount of an active compound (compound D, F, G, or H) was added. Finally, 10 mL of acetic acid at 1% m/v was also added. The suspension obtained was vigorously stirred with a spatula for 1 min and transferred to a glass Petri dish with a 5 cm diameter. The mixture was allowed to dry for three days, and then a solid membrane was peeled from the dish. To increase the mechanical stiffness of the membrane, rectangular pieces of 40 by 20 mm were cut and subjected to an irradiation step to enhance the crosslinking inside the membrane. Without this step, the membranes were not stable for long periods inside water. This step was also supposed to help keep the active compounds inside the membranes.

### 2.7. Instrumentation and Analysis

The metal concentrations in aqueous phase were measured using an ICP-MS equipment installed in a clean room, class 5, at the Centro de Ciências e Tecnologias Nucleares (C^2^TN). The ICP-MS is equipped with a quadrupole system ELAN DRC-e (Axial Field Technology) from PerkinElmer^®^ Sciex. This equipment is composed of a sample introduction system, which contains a concentric PFA nebulizer and a cyclonic spray chamber with a Peltier cooled PC3. The sampler and skimmer cones are made of Ni. Quantitative analysis was conducted using a 10 µg/mL, STD ICP-MS MULTI ELEMENT CAL solution PerkinElmer^®^, Inc. Pure Plus, USA), and the calibration method used was with external standardization. The samples and STD 2 were diluted with 5% HNO_3_ solution from 65% HNO_3_ purified using sub-boiling distillation (bi-distilled) and diluted with Milli-Q water (18.2 MΩ cm) obtained with an ultra-pure water purification system (Merck Millipore, S.A.S., Molsheim, France). This system ensured that the initial pH of the water was 6.998 and the amount of salts was minimal. The isotopes ^139^La, ^140^Ce, ^146^Nd, ^147^Sm, ^157^Gd, ^163^Dy, ^166^Er, and ^172^Yb were used to assess elemental concentrations, and ^103^Rh was used for internal standardization to correct for instrumental drifts and non-spectral interferences.

Gamma irradiations were performed in a cobalt-60 experimental irradiator (model Precisa 22, Graviner Lda, UK, 1971) located in the Ionizing Radiation Facilities (IRIS) at the Centro de Ciências e Tecnologias Nucleares (C^2^TN), at a dose rate of 0.5 kGy·h^−1^. Routine Amber Perspex dosimeters from Harwell (Oxford, UK) were used for monitoring the membranes’ absorbed dose. In this study, the membranes were gamma-irradiated at 5 and 10 kGy. These relatively low doses were used in order to not influence the activity of the active compounds inside the membranes toward the metals. Higher doses could induce more crosslinking but could also degrade the active compounds.

### 2.8. Hydration Capacity of Membranes

The hydration capacity or hydration behavior of a polymeric support has particular importance when it is to be used for long periods in water. The PVDF membranes used in this study were essentially hydrophobic in nature, but the chitosan-based membranes were rather hydrophilic in structure. The amount a membrane can absorb is quite relevant. If it is higher, an easier exchange with the surrounding solution can be attained. However, a very high value can modify the structure of the membrane and some of its mechanical properties. In the present study, the chitosan-based membrane without being irradiated absorbed a huge amount of water, but it ended up in a gelatinous form with very low mechanical resistance, which would prevent its use in real-life situations. Hydration capacity was studied for the chitosan-based membranes by leaving the membranes in water for 24 h and weighing their mass at the beginning and at the end. For the PVDF membranes, which were hydrophobic, this measurement made no sense and was not performed.

### 2.9. Stability of Absorbed Active Compounds Inside PVDF Membranes

After the impregnation and drying procedure, the PVDF membranes with immobilized active compounds (or mixtures) were placed separately in 250 mL Erlenmeyer flasks with Milli-Q water (100 mL), under orbital shaking for 1 h at room temperature. After this period, the membranes were taken out from the solution. Excess solution was removed with absorbent paper, and the remaining impregnated membranes were stored overnight in an air desiccator with silica gel. To determine the amount of ionic liquid (or mixture) that remained, the membranes were weighed before and after the procedure.

### 2.10. Extraction Experiments

For the extraction experiments, an initial lanthanide solution with 1 × 10^−4^ M concentration of Ln^3+^ ([Ce(NO_3_)_3_(H_2_O)_6_], [Nd(NO_3_)_3_(H_2_O)_6_], [Sm(NO_3_)_3_(H_2_O)_6_], [Gd(NO_3_)_3_(H_2_O)_6_], [Dy(NO_3_)_3_(H_2_O)_5_], [Er(NO_3_)_3_(H_2_O)_5_], and [Yb(NO_3_)_3_(H_2_O)_5_] each was prepared by dissolving the appropriate hydrated lanthanide nitrates in Milli-Q water (see above).

To study the ability of the prepared membranes to achieve the goals of this study (see final paragraph of the Introduction), all studied membranes were placed in large volumes (ca. 100 mL) of the previous solution under orbital shaking for around 24 h at room temperature. After this procedure, the membranes were taken out from the solution, excess solution was removed with absorbent paper, and the lanthanides retained in the membranes were recovered using back extraction. This was performed by putting the membranes in a small can with 1 mL of 0.5M HNO_3_ solution, which was shaken in a vortex mixer for 15 min at 2000 rpm, and then the final solution was separated and analyzed using ICP-MS.

The percentage of extraction (%E) was calculated by dividing the amount of the lanthanides extracted by the membranes and deposited on the final 1 mL solution relative to the initial amount of each metal in the initial aqueous solution (Equation (1)):

(1) $$\%\;\mathrm{extraction}=\frac{{\mathrm{Ln}}_{\mathrm{ext}}^{3+}}{{\mathrm{Ln}}_{\mathrm{aq}}^{3+}}\times 100$$

where ${\mathrm{Ln}}_{\mathrm{aq}}^{3+}$ is the molar lanthanide amount in the initial aqueous solution before extraction, and ${\mathrm{Ln}}_{\mathrm{ext}}^{3+}$ is the final molar amount in the final solution.

## 3. Results and Discussion

### 3.1. Hydration Capacity of Membranes

**Figure 1:**
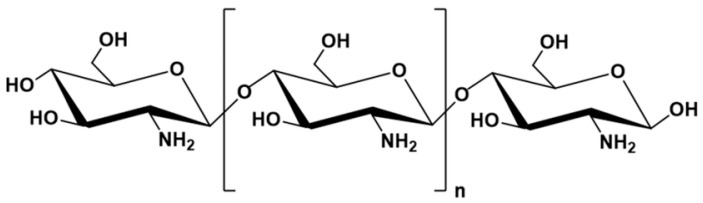
A schematic of chitosan structure.

Chitosan is a linear polysaccharide composed of D-glucosamine and N-acetyl-D-glucosamine units (Figure 1). It is obtained by treating chitin with an alkaline substance, thereby promoting deacetylation. When preparing chitosan membranes, several chains of the material are entangled with each other, but there is no chemical bond between them. Gamma radiation promotes the formation of radicals (namely in the OH function) that leads to the establishment of chemical bonds among the chains. This process improves the mechanical properties and stability in water of membranes. However, if this process is taken too far, the amount of new radiation-induced bonds can be high enough to significantly reduce the porosity of the membranes. For an application like the one that is intended, the existence of high porosity is very important.

The dose chosen for the irradiation of the membranes was inspired by previous work in the literature, aiming to be high enough to improve the mechanical properties of the membranes (namely their stability in water) while keeping their porosity [27]. The chitosan membranes without being submitted to irradiation present a percentage mass gain of around 6000% after submerged in water for 24 h. This is a huge increase in their masses. After an additional 24 h, some of them begin to lose their physical consistency and, in some cases, it is no longer possible to remove them in one piece from water. The inclusion of an irradiation step solves this problem due to an increase in crosslinking but at the expense of some decrease in the ability to absorb water. For the irradiated chitosan membranes (both at 5 and 10 kGy doses of irradiation), the mass increment in water is presented in Table 1. It can be observed that the values decrease a lot when compared with the non-irradiated membranes, but still significant and on the same order of magnitude at both irradiation doses.

### 3.2. Stability of Absorbed Active Compounds in PVDF Membranes

**Figure 2:**
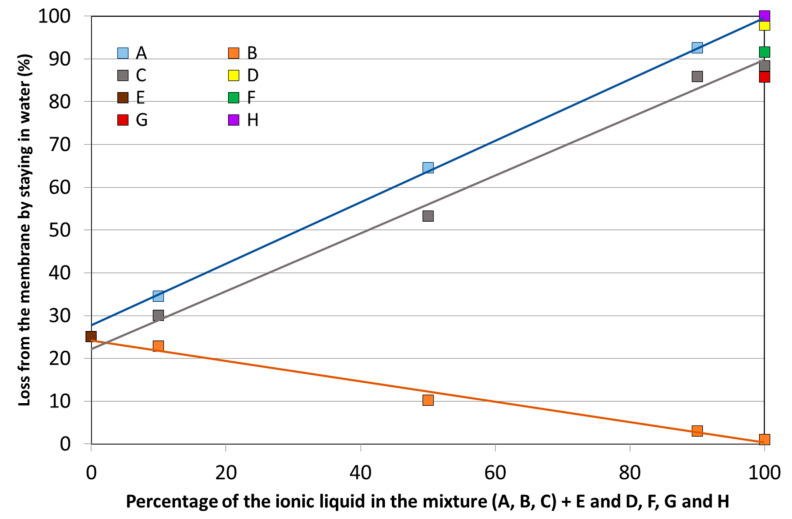
Stability of the compounds or mixtures of compounds in the PVDF membranes after 24 h in water with agitation. A value of 100% means that all the active compounds are lost to the solution, and 0% means that all of them remains in the membrane.

A first step after the soaking of PVDF membranes is to check if active compounds are kept inside the membranes after long periods of being immersed in water. This is very important if these membranes are intended to stay for a long time in water. For that purpose, several membranes were prepared with all compounds in the study (A, B, C, D, E, F, G, and H). In addition, membranes with A, B, C, and D (the four ionic liquids used in this study) mixed with tributyl phosphate (compound E) in 1:9, 5:5, and 9:1 proportion were also prepared. The idea was to check, according to the literature data [28], that some effect on the stabilization of ionic liquids inside the membranes could be achieved by the addition of tributyl phosphate. The results from the combinations mentioned above are presented in Figure 2, with the intention of representing all of them in only one figure. The pure compounds (with the exception of tributyl phosphate) have their values presented on the right side of the figure. Tributyl phosphate is presented on the left side, and the membranes with mixtures of ionic liquids and tributyl phosphate are presented in the middle of the figure. With the exception of compound B—trihexyltetradecylphosphonium bis(trifluoromethylsulfonyl)amide (which stays essential inside all membranes) and compound E—tributyl phosphate (where 25% remains inside the membranes), a great amount of all the other compounds is lost to the aqueous solution (between 85 and 98%). The mixtures of ionic liquids with compound E do not have any synergistic effect, and the verified loss is only the weighted average of the losses of the two compounds under analysis.

The reason that only the two compounds that possess phosphorous in their composition remain in a significant amount inside the PVDF (polyvinylidene difluoride) membranes could be linked to the possibility of their chemical reaction with the fluorinated backbone of the membranes [29]. This reaction makes them very stable inside the membrane but also results in a loss of their coordination capability to metals and, consequently, their usefulness as extractants (see below).

### 3.3. Extraction Results of the Studied Membranes

**Figure 3:**
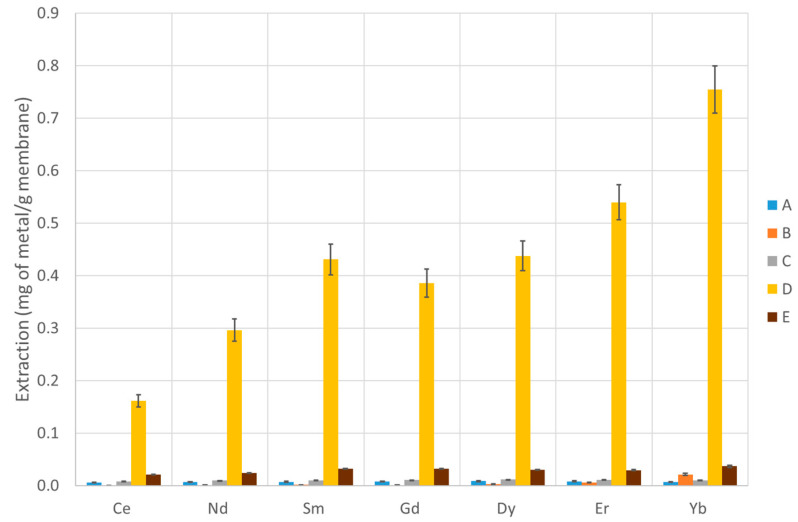
Extraction of each metal under study using the PVDF membranes with ionic liquids and tributyl phosphate compounds.

**Figure 4:**
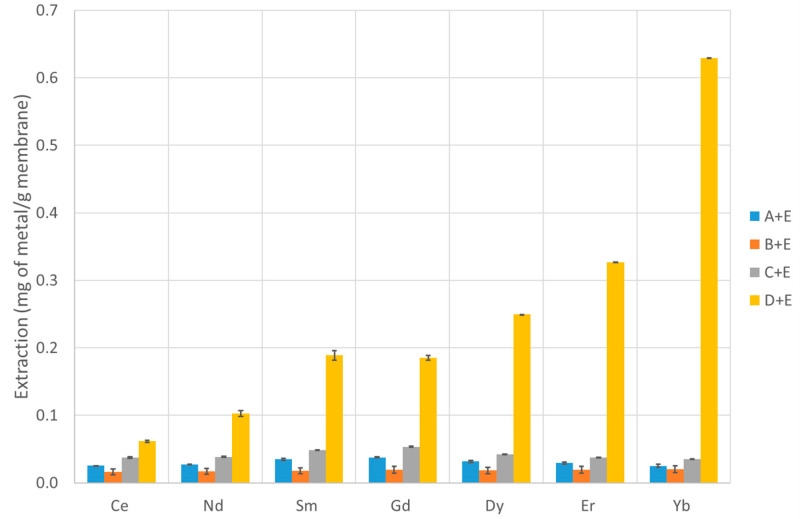
Extraction of each metal under study using the PVDF membranes with mixtures of ionic liquids and tributyl phosphate.

**Figure 5:**
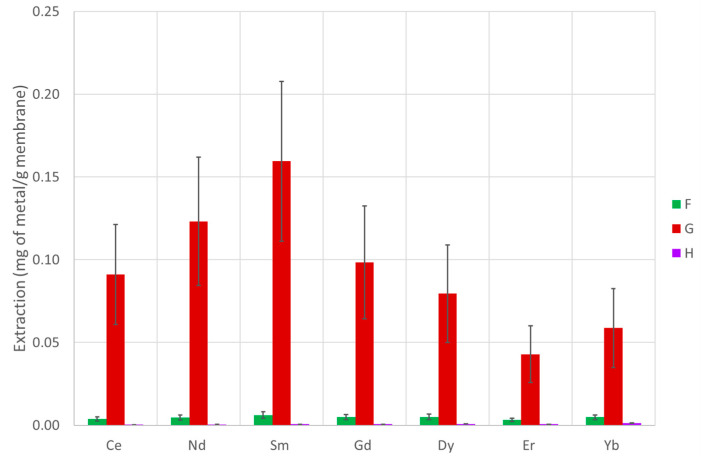
Extraction of each metal under study using the PVDF membranes with sucrose/carboxylic acid compounds.

**Figure 6:**
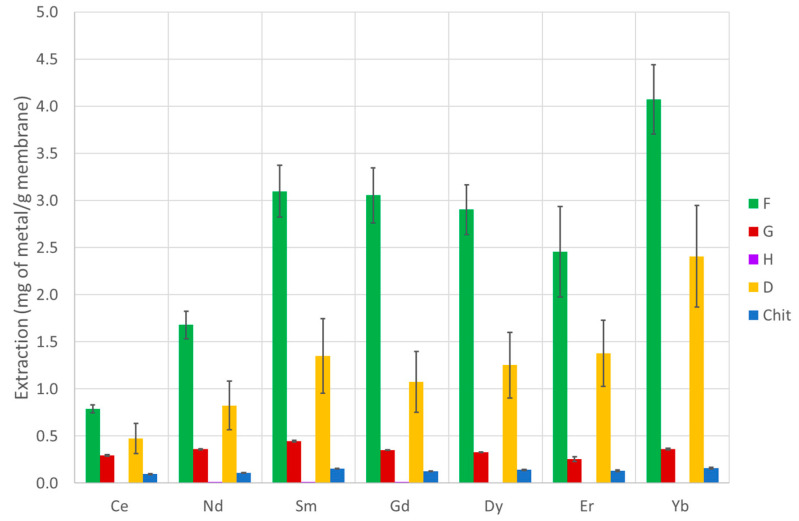
Extraction of each metal under study using the chitosan-based membranes with several active compounds irradiated at 5 kGy with a dose rate (DR) of 0.5 kGy h^−1^.

**Figure 7:**
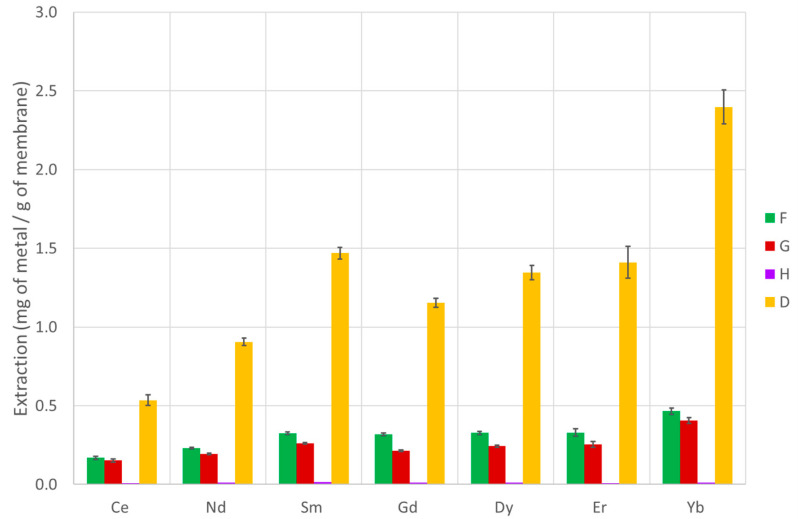
Extraction of each metal under study using the chitosan-based membranes with several active compounds irradiated at 10 kGy (DR = 0.5 kGy.h^−1^).

The extraction capability of several PVDF-supported liquid membranes and chitosan-based membranes were tested according to the procedure described in Section 2.10, and the efficiency over seven lanthanide metals (covering the entire series) was determined. The results are presented in Table 2 and Table 3 and Figure 3, Figure 4 and Figure 5 (PVDF membranes), and Table 4 and Table 5 and Figure 6 and Figure 7 (chitosan-based membranes).

The first tables of each type of membranes (mg of metal extracted by g of membrane—Table 2 and Table 4) are important to assess their capability in retaining lanthanides, while the second tables (Table 3 and Table 5) show the percentage of extraction as defined earlier. The data are not normalized for the membranes’ masses. However, it should be stressed that, since the initial solution has a volume of 100 mL and the final back-extracted solution has a volume of 1 mL, when the value of percentage extracted is higher than 1%, this means that the concentration in the final 1 mL is higher than in the initial solution. This reflects the metal concentration effect that these membranes are capable of providing, which is a fundamental parameter for the purpose of this study. When that happens, it could be referred that there is a concentration effect.

In the PVDF membranes, the amounts of active compounds are quite low in most cases; thus, it is not expected that the membrane performance would be high. However, the case of IL oxamate [C_4_mim][DEHOX] (D) deserves a note. Even though only 5% of the compound D mass initially absorbed by the membrane is retained in the PVDF after a long period of water immersion, this small amount is enough to remove about 2.5% of Yb existing in the initial solution (reaching 0.75 mg of Yb per gram of membrane). For all the lanthanides (except Ce), a concentration effect is noticed (Table 3). Thus, even with this small amount of IL loaded in the membrane, a concentration effect of 2.5 times for Yb is achieved. The suitability of the ionic liquid D as a good agent to extract lanthanides is also confirmed with the chitosan membranes, but this is not surprising since the literature data on liquid–liquid extraction already show that it is a good lanthanide extractor [18]. Despite the lower values of extraction for most of the membranes, and not being one of the main objectives of the study, some differences are also observed in the selectivity of the membranes along the lanthanide series. While the membranes with compounds A, C, and E show very low selectivity, the membranes with ILs [P_66614_][NTf_2_] (B) and [C_4_mim][DEHOX] (D) present a noticeable selectivity along the series. The separation factor is defined as the percentage of extraction of one metal divided by the percentage of extraction of another metal and measures the ability of a system to separate two metals. The membrane with compound B presents a Yb/Ce separation factor of 51, and the one with compound D presents a value of 4.7.

The mixture of ionic liquids with tributyl phosphate (E) shows no improvement when compared with the isolated ionic liquids (Figure 3 and 4). Nevertheless, the ionic liquid D is still the best one with small concentration effects for Er and Yb. Curiously, the separation factor increases to 10 for the membrane with the mixture of compounds D + E (1-Butyl-3-methylimidazolium-di-(2-ethylhexyl)-oxamate and tributyl phosphate) when compared with the one with only compound D. None of the other PVDF membranes loaded with other compounds present a comparable result, not even the membranes with compound B or E that remains in a significant amount inside the membranes.

With the chitosan-based membranes, the panorama is radically distinct. Almost all the membranes present a high extraction for all lanthanides when expressed as mg of metal extracted by gram of membrane, and most of them also have the capability to obtain a final solution after back extraction that is more concentrated than the initial solution. The exceptions are the membranes possessing only chitosan and the ones synthesized with compound H (sucrose/succinic acid). All the others present a highly positive behavior. A general trend is that the extraction of heavy lanthanides is slightly higher than that of lighter ones. The membrane that presents a better result is the one with compound F (sucrose/citric acid) irradiated at 5 kGy, which achieves a concentration factor bigger than 13 (more than one order of magnitude) and is capable of extracting more than 4 mg of Yb per gram of membrane, achieving a total of more than 18 mg of lanthanides per gram of membrane. Another interesting point is that for the membranes with IL [C_4_mim][DEHOX] (D), the membranes irradiated at 10 kGy are slightly better extractants when compared to the ones irradiated at 5 kGy. However, the opposite is seen for the membranes with citric acid/sucrose (F), while for the membranes with sucrose/myristic acid (G), the values are quite similar in both cases. Although this is not one of the main goals of the study, it should be noticed that the membranes with compounds F (sucrose/citric acid) and D (1-Butyl-3-methylimidazolium-di-(2-ethylhexyl)-oxamate) present a Yb/Ce separation factor of 5.1 and 5.1 for the membranes irradiated at 5 kGy and 2.8 and 4.5 for the membranes irradiated at 10 kGy, respectively. It should be stressed that the carboxylic acids used to prepare compounds F, G, and H present some differences: F is a small-chain compound with three carboxylic functions plus an OH function (citric acid), G is a long chain with only one carboxylic function at one of the ends (myristic acid), and H has two carboxylic functions at both ends of a small chain (succinic acid). Only a more in-depth study of the structure of the carboxylic acids and ionic liquids in the chitosan membrane structures could elucidate these differences; however, as a first attempt, the presence of a large number of OH groups in compound F (sucrose/citric acid) could be responsible for the more sensitive response of this particular membrane toward the radiation dose.

## 4. Conclusions

In this work, two approaches were investigated for extracting lanthanides from low-concentration aqueous solutions: (1) the use of PVDF membranes loaded with active compounds or their mixtures, and (2) the use of synthesized chitosan-based membranes that already contained active compounds in their composition. The underlying idea was to obtain a means of recovering metals, i.e., lanthanides, in very dilute aqueous solutions, since this is the reality in many wastewaters and conventional extraction methods (e.g., liquid–liquid separation) are not feasible for very low concentrations. For this purpose, several membranes were prepared, and extraction studies were performed with simulated 10^−4^ M aqueous solutions of lanthanides. The prepared solutions contained seven lanthanides (Ce, Nd, Sm, Gd, Dy, Er, and Yb), covering the whole series of them. All analytical measurements of lanthanides in the solutions were performed using ICP-MS.

The first approach with the PVDF membranes loaded with a variety of commercial or synthesized ionic liquids, their mixture with tributyl phosphate, or some Lewis adducts showed quite poor results. Only one family of the membranes (the one loaded with compound D—([C_4_mim][DEHOX]) presented some positive results. Although the fact that other membranes had such negative results was something of a surprise, the relatively good results obtained with oxamate ionic liquid (D) were not a surprise as this ionic liquid had already demonstrated good capability for extracting lanthanides in liquid–liquid extraction [18]. However, the best obtained result only led to a concentration factor of 2.5 times for Yb.

A completely different panorama was obtained with the chitosan-based synthesized membranes. Almost all of them had a significant capability to extract lanthanides from low-concentration aqueous solutions, and it is clear that the observed capability was not due to the backbone chitosan structure (since membranes with no active compound showed no extraction potential) but rather to the compounds added to them. Again, the membrane synthesized with the ionic liquid D [18] showed a concentration power of more than 6 times, both in membranes irradiated at 5 or 10 kGy. Additionally, the membranes made with sucrose/myristic acid presented a very good result, attaining a concentration performance higher than two for all the membranes. However, the best result was obtained with the membranes containing sucrose/citric acid and irradiated at 5 kGy. These membranes could achieve a concentration factor higher than 13, which is very encouraging for further study of this family of membranes. These membranes achieved a capacity of extraction of 18 mg of lanthanides per gram of membrane, which shows the good prospects of their use for the effective recovery of lanthanides in wastewater.

Some of the membranes showed no discrimination among the lanthanides, but the majority of them displayed a pattern of extracting preferentially heavy lanthanides when compared with lighter ones. The chitosan membranes with compounds F (sucrose/citric acid) and D (1-Butyl-3-methylimidazolium-di(2-ethylhexyl)-oxamate) presented a Yb/Ce separation factor of 5.1 and 5.1 for membranes irradiated at 5 kGy and 2.8 and 4.5 for membranes irradiated at 10 kGy, respectively.

To the best of our knowledge, this is the first time that chitosan membranes were used to extract lanthanides from low-concentration solutions. These membranes are easily synthesized at a very low cost, and further studies are already planned, either with new compositions or using other geometries and extraction systems.

## Figures and Tables

**Table 1:** Chitosan membranes’ water uptake.

Membrane	Average Mass Increment after 24 h in Water (%) ^1^
Chitosan not irradiated	6127
Chit(D)-5 kGy	88
Chit(F)-5 kGy	1966
Chit(G)-5 kGy	17
Chit(H)-5 kGy	130
Chit(D)-10 kGy	123
Chit(F)-10 kGy	1701
Chit(G)-10 kGy	81
Chit(H)-10 kGy	134

^1^ Several repetitions lead to an average dispersion of around 5%.

**Table 2:** Extraction of lanthanides using the PVDF membranes (mg of metal/g of membrane).

Membrane	Ce	Nd	Sm	Gd	Dy	Er	Yb
PVDF(A)	0.0056 ± 0.0004	0.0067 ± 0.0007	0.0071 ± 0.0008	0.0074 ± 0.0008	0.0083 ± 0.0009	0.0079 ± 0.0008	0.0063 ± 0.0007
PVDF(B)	0.0004 ± 0.0001	0.0006 ± 0.0001	0.0012 ± 0.0002	0.0009 ± 0.0001	0.0023 0.0003	0.0053 ± 0.0005	0.0210 ± 0.0019
PVDF(C)	0.0080 ± 0.0001	0.0091 ± 0.0001	0.0096 ± 0.0001	0.0101 ± 0.0001	0.0113 ± 0.0001	0.0107 ± 0.0001	0.0095 ± 0.0002
PVDF(D)	0.1608 ± 0.0115	0.2960 ± 0.0213	0.4308 ± 0.0292	0.3852 ± 0.0269	0.4374 ± 0.0286	0.5394 ± 0.0333	0.7541 ± 0.449
PVDF(E)	0.0212 ± 0.0005	0.0235 ± 0.0003	0.0319 ± 0.0002	0.0317 ± 0.0003	0.0295 ± 0.0006	0.0293 ± 0.0007	0.0364 ± 0.0015
PVDF(A + E)	0.0254 ± 0.00001	0.0271 ± 0.0006	0.0346 ± 0.0013	0.0377 ± 0.0011	0.0316 ± 0.0013	0.0291 ± 0.0016	0.0251 ± 0.0027
PVDF(B + E)	0.0162 ± 0.0041	0.0169 ± 0.0043	0.0177 ± 0.0046	0.0192 ± 0.0049	0.0182 ± 0.0047	0.0196 ± 0.0051	0.0201 ± 0.0052
PVDF(C + E)	0.0375 ± 0.0011	0.0382 ± 0.0008	0.0485 ± 0.0005	0.0532 ± 0.0007	0.0421 ± 0.0004	0.0376 ± 0.0003	0.0352 ± 0.0001
PVDF(D + E)	0.0615 ± 0.0014	0.1025 ± 0.0042	0.1887 ± 0.0073	0.1851 ± 0.0039	0.2494 ± 0.0001	0.3268 ± 0.0004	0.6291 ± 0.0004
PVDF(F)	0.0037 ± 0.0013	0.0046 ± 0.0015	0.0062 ± 0.0020	0.0049 ± 0.0016	0.0051 ± 0.0017	0.0032 ± 0.0010	0.0048 ± 0.0015
PVDF(G)	0.0911 ± 0.0302	0.1232 ± 0.0388	0.1594 ± 0.0484	0.0983 ± 0.0341	0.0795 ± 0.0294	0.0429 ± 0.0170	0.0587 ± 0.0238
PVDF(H)	0.0004 ± 0.0001	0.0005 ± 0.0001	0.0007 ± 0.0001	0.0006 ± 0.0001	0.0008 ± 0.0001	0.0007 ± 0.0002	0.0014 ± 0.0003

**Table 3:** Percentage extraction of lanthanides using the PVDF membranes (% of existing metal in the initial solution).

Membrane	Ce	Nd	Sm	Gd	Dy	Er	Yb
PVDF(A)	0.025 ± 0.002	0.029 ± 0.001	0.029 ± 0.001	0.029 ± 0.001	0.032 ± 0.001	0.029 ± 0.001	0.023 ± 0.001
PVDF(B)	0.002 ± 0.001	0.002 ± 0.001	0.005 ± 0.001	0.003 ± 0.001	0.008 0.002	0.019 ± 0.004	0.073 ± 0.013
PVDF(C)	0.037 ± 0.007	0.041 ± 0.008	0.042 ± 0.009	0.042 ± 0.009	0.046 ± 0.010	0.042 ± 0.009	0.036 ± 0.009
PVDF(D)	0.702 ± 0.111	1.256 ± 0.199	1.754 ± 0.264	1.500 ± 0.232	1.647 ± 0.240	1.974 ± 0.273	2.667 ± 0.357
PVDF(E)	0.097 ± 0.006	0.104 ± 0.005	0.136 ± 0.001	0.129 ± 0.001	0.116 ± 0.002	0.112 ± 0.003	0.134 ± 0.008
PVDF(A + E)	0.104 ± 0.007	0.108 ± 0.002	0.132 ± 0.001	0.138 ± 0.002	0.111 ± 0.002	0.100 ± 0.004	0.083 ± 0.012
PVDF(B + E)	0.073 ± 0.039	0.074 ± 0.039	0.074 ± 0.040	0.077 ± 0.041	0.071 ± 0.038	0.074 ± 0.040	0.073 ± 0.040
PVDF(C + E)	0.160 ± 0.004	0.158 ± 0.002	0.192 ± 0.002	0.202 ± 0.001	0.155 ± 0.002	0.134 ± 0.002	0.122 ± 0.004
PVDF(D + E)	0.271 ± 0.022	0.439 ± 0.051	0.775 ± 0.088	0.726 ± 0.056	0.946 ± 0.034	1.204 ± 0.040	2.241 ± 0.082
PVDF(F)	0.006 ± 0.003	0.007 ± 0.004	0.009 ± 0.005	0.007 ± 0.004	0.007 ± 0.004	0.004 ± 0.002	0.006 ± 0.003
PVDF(G)	0.188 ± 0.132	0.247 ± 0.166	0.306 ± 0.199	0.181 ± 0.133	0.142 ± 0.110	0.075 ± 0.062	0.099 ± 0.083
PVDF(H)	0.001 ± 0.000	0.001 ± 0.001	0.002 ± 0.000	0.001 ± 0.000	0.002 ± 0.001	0.001 ± 0.000	0.003 ± 0.001

**Table 4:** Extraction of lanthanides using the chitosan membranes (mg of metal/g of membrane).

Membrane	Ce	Nd	Sm	Gd	Dy	Er	Yb
Chit(F)-5 kGy	0.79 ± 0.04	1.68 ± 0.15	3.10 ± 0.28	3.05 ± 0.29	2.90 ± 0.26	2.46 ± 0.48	4.07 ± 0.37
Chit(G)-5 kGy	0.30 ± 0.01	0.36 ± 0.01	0.45 ± 0.01	0.35 ± 0.01	0.33 ± 0.01	0.26 ± 0.03	0.36 ± 0.01
Chit(H)-5 kGy	0.01 ± 0.01	0.01 ± 0.01	0.02 ± 0.01	0.01 ± 0.01	0.01 ± 0.01	0.01 ± 0.01	0.01 ± 0.01
Chit(D)-5 kGy	0.47 ± 0.16	0.82 ± 0.26	1.35 ± 0.39	1.07 ± 0.33	1.25 ± 0.35	11.38 ± 0.35	2.41 ± 0.54
Chitosan-5 kGy	0.10 ± 0.01	0.11 ± 0.01	0.15 ± 0.01	0.12 ± 0.01	0.14 ± 0.01	0.13 ± 0.01	0.16 ± 0.01
Chit(F)-10 kGy	0.17 ± 0.01	0.23 ± 0.01	0.33 ± 0.01	0.32 ± 0.01	0.33 ± 0.01	0.33 ± 0.02	0.47 ± 0.02
Chit(G)-10 kGy	0.15 ± 0.01	0.19 ± 0.01	0.26 ± 0.01	0.21 ± 0.01	0.24 ± 0.01	0.26 ± 0.02	0.40 ± 0.02
Chit(H)-10 kGy	0.01 ± 0.01	0.01 ± 0.01	0.01 ± 0.01	0.01 ± 0.01	0.01 ± 0.01	0.01 ± 0.01	0.01 ± 0.01
Chit(D)-10 kGy	0.53 ± 0.03	0.91 ± 0.02	1.47 ± 0.04	1.15 ± 0.03	1.35 ± 0.05	1.41 ± 0.10	2.40 ± 0.11

**Table 5:** Percentage extraction of lanthanides using the chitosan membranes (% of existing metal in the initial solution).

Membrane	Ce	Nd	Sm	Gd	Dy	Er	Yb
Chit(F)-5 kGy	3.22 ± 0.32	6.62 ± 0.18	11.71 ± 0.25	11.02 ± 0.08	10.15 ± 0.19	8.16 ± 1.63	13.37 ± 0.24
Chit(G)-5 kGy	3.09 ± 0.49	3.65 ± 0.28	4.34 ± 0.34	3.25 ± 0.30	2.98 ± 0.35	2.22 ± 0.20	3.06 ± 0.49
Chit(H)-5 kGy	0.098 ± 0.022	0.116 ± 0.004	0.148 ± 0.008	0.107 ± 0.020	0.097 ± 0.029	0.075 ± 0.047	0.090 ± 0.036
Chit(D)-5 kGy	1.58 ± 1.26	2.65 ± 1.99	4.16 ± 2.95	3.17 ± 2.31	3.56 ± 2.44	3.80 ± 2.44	6.37 ± 3.72
Chitosan-5 kGy	0.279 ± 0.036	0.317 ± 0.016	0.417 ± 0.021	0.324 ± 0.016	0.355 ± 0.024	0.322 ± 0.047	0.381 ± 0.034
Chit(F)-10 kGy	1.472 ± 0.189	1.961 ± 0.100	2.652 ± 0.134	2.481 ± 0.121	2.462 ± 0.169	2.404 ± 0.348	3.292 ± 0.294
Chit(G)-10 kGy	1.942 ± 0.250	2.425 ± 0.124	3.114 ± 0.158	2.445 ± 0.120	2.677 ± 0.183	2.740 ± 0.396	4.195 ± 0.375
Chit(H)-10 kGy	0.098 ± 0.013	0.112 ± 0.006	0.141 ± 0.007	0.105 ± 0.005	0.097 ± 0.007	0.083 ± 0.012	0.097 ± 0.009
Chit(D)-10 kGy	2.037 ± 0.262	3.351 ± 0.172	5.216 ± 0.264	3.918 ± 0.192	4.424 ± 0.303	4.504 ± 0.651	7.398 ± 0.662

## Data Availability

Data is contained within the article.
